# Coordinated Collaboration and Nonverbal Social Interactions: A Formal and Functional Analysis of Gaze, Gestures, and Other Body Movements in a Contemporary Dance Improvisation Performance

**DOI:** 10.1007/s10919-019-00313-2

**Published:** 2019-07-22

**Authors:** Vito Evola, Joanna Skubisz

**Affiliations:** BlackBox Project, ICNOVA, FCSH, Universidade Nova de Lisboa, Avenida de Berna, 26 (EID-2.14), 1069-061 Lisbon, Portugal

**Keywords:** Collaboration and decision-making, Dance improvisation and intuition, Nonverbal interactions, Social cognition, Body movements, Gaze and attention

## Abstract

This study presents a microanalysis of what information performers “give” and “give off” to each other via their bodies during a contemporary dance improvisation. We compare what expert performers and non-performers (sufficiently trained to successfully perform) do with their bodies during a silent, multiparty improvisation exercise, in order to identify any differences and to provide insight into nonverbal communication in a less conventional setting. The coordinated collaboration of the participants (two groups of six) was examined in a frame-by-frame analysis focusing on all body movements, including gaze shifts as well as the formal and functional movement units produced in the head–face, upper-, and lower-body regions. The Methods section describes in detail the annotation process and inter-rater agreement. The results of this study indicate that expert performers during the improvisation are in “performance mode” and have embodied other social cognitive strategies and skills (e.g., endogenous orienting, gaze avoidance, greater motor control) that the non-performers do not have available. Expert performers avoid using intentional communication, relying on information to be inferentially communicated in order to coordinate collaboratively, with silence and stillness being construed as meaningful in that social practice and context. The information that expert performers produce is quantitatively less (i.e., producing fewer body movements) and qualitatively more inferential than intentional compared to a control group of non-performers, which affects the quality of the performance.

## Introduction

When watching a performed improvisation, where creativity emerges from performers’ coordinated collaboration, it may seem remarkable how the performers know what to do and when, and without it seeming haphazard. Performers pick up cues from what is happening around them and make appropriate decisions in the moment, with their actions being a performance the entire time. In fact, a lay person might say the better the improvisation, the less it appears to be “improvised”. For various choreographers and dancers, the ultimate key in the performer’s decision-making process is “intuition” (e.g., Mary O’Donnell, João Fiadeiro; in da Silva 2010, pp. 21, 51). Choreographer and researcher Jonathan Burrows in his *A Choreographer’s Handbook* posits various maxims or principles (one being “follow your intuition”) so as to avoid being overwhelmed in the creative process and being “free to do what you do best, which is to be intuitive” (Burrows 2010, p. 2); another book by choreographer Darla Johnson is subtitled *Intuition and Improvisation in Choreography* (2012). Intuition, nevertheless, as a “folk psychological notion” (Cosmides and Tooby 2008, p. 844), is too imprecise to have descriptive or explanatory value for cognitive science. Just how collaborative information is construed and processed in language-absent, face-to-face interactions like improvisations remains largely to be explored.

This study presents a microanalysis of the phenomenology of coordinated collaboration in a less typical social context of joint activity, which has no regulated turns in the traditional sense and is linguistically independent.^1^ The improvisation exercise analyzed below is practiced by both expert performers and non-performers, often with equally creative outcomes. Nonetheless, the quality of the activity “feels” different depending on who performs it: Unsurprisingly, experts tend to exhibit both greater motor coordination (defined as producing movements that are kinematically and kinetically more coordinated or “well timed, smooth, and efficient with respect to the intended goal”; Bar-Haim et al. 2013, p. 5) as well as greater social coordination (e.g., disambiguated turn-taking), making their movements appear to be pre-coordinated. Expert performers train to use their bodies in a way that is destined “for show”, implying that they have a different perspective and handling of their bodies than non-performers (cf. “impression management”: Goffman 1959). They have more practice as well as potentially different socio-cognitive skills than non-performers in solely using their bodies (i.e., without language) within a controlled environment to communicate intentions, whereas non-performers may need to revert to more familiar skill-sets (such as those used in everyday human dialogue interactions). The performers’ embodiment of these cognitive schemata and the description of their behavior in coordinated collaboration in a dance improvisation is the focus of this study.

This study is the continued development of previous research (Evola et al. 2016), analyzing silent coordinated collaboration in the context of contemporary dance improvisation. We compare what expert performers and non-performers (sufficiently trained to successfully perform the improvisation exercise) do with their bodies during a silent, multiparty improvisation exercise in order to identify any differences and to provide insight into nonverbal communication in less conventional settings. Because of the silent nature of the improvisation exercise, the bodies bear the burden of conveying socially coordinating and collaborating information. Unlike other studies on human collaboration and coordination, typically concentrating on turn-taking in language, the data for this study were collected in a less traditional context, although just as naturally occurring, namely in a creative artistic setting. In fact, the present study intends to fill the gap in the existing literature by offering the first-of-its-kind, fine-grained analysis of nonverbal behavior in multiparty group interactions (as opposed to more frequent dyadic interactions) where speech is accessible but prohibited. Moreover, we offer a cognitive-semiotic analysis comparing expert versus non-expert pragmatic behavior under similar conditions, contributing to the growing field of expertise studies in cognitive psychology (e.g., Chi et al. 1988) as well as providing empirical data in the context of social interactions in artistic settings, a field of inquiry proving to be productive in recent times (see review below). Through a qualitative and quantitative analysis, we investigate whether these experts use the same strategies described in the literature to socially coordinate the group’s actions and interactions, or if they have in some way learned to use their bodies differently for the sake of performance. We distinguish the communication of information into two types: intentional (“given”) or inferential (“given off”; Goffman 1963). The overarching hypothesis is that, unlike non-performers, the information generated by expert performers will be quantitatively less (i.e., producing fewer body movements), and this information will be of a more inferential than intentional nature.

### Dance Improvisation and J. Fiadeiro’s “Real Time Composition” Game

The use of improvisation, or of unplanned movements, is one of the elements that characterizes contemporary dance, allowing performers to express their feelings and inspirations freely at a given moment, with the potential of both performers and audience being “taken by surprise” in and by the process (Albright and Gere 2003). Many contemporary collaborative choreographies are developed using improvised movement material that arises from exercises in the dance studio (Mason 2009). In these proto-performances, how the dancers know what to do and when, without it seeming random or chaotic, is not the focus of the choreographers and performers themselves, but it will be what our inquiry aims at explicating.

In the domain of the performing arts such as dance, there are a variety of improvisation forms, techniques, and methods. This present study focuses on the Real Time Composition method (*Composição em Tempo Real*, or CTR), developed in 1995, and since then systematized thanks to various collaborations and continued research, by internationally renowned Portuguese contemporary choreographer João Fiadeiro (see Fiadeiro 2007; Jürgens and Fernandes 2017; Jürgens et al. 2016). Despite more recent methodological modifications to the CTR (see Fiadeiro 2017), we present a snapshot of our understanding of it as it was in 2015. Fiadeiro, one of the pioneers of the *Nova Dança Portuguesa*, developed an exercise in this improvisation method called the “CTR Game” (henceforth: the Game) in order to provide choreographers and dancers a methodological tool for creating material for their artistic works. With its strong Postmodern influence, CTR aims at exploring everyday movements as well as more traditional dance techniques. This genre of dance performance, commonplace in the field of dance, may be considered “unconventional” by the layperson, as it incorporates informal actions like moving around on stage with adhesive tape or making drawings with one’s body as performance. Fiadeiro’s method of Real Time Composition, as is common in contemporary dance, focuses on the creative process rather than on the product. From what creatively and collaboratively emerges throughout the Game, innovative ideas are generated for compositions and performances destined for the stage.

The CTR method’s fundamental exercise is “playing the Game”: With no prior choreographic or dramaturgic instructions, practitioners are free to take turns in performing (or not) using their bodies and props, all the time building on each other’s actions to form “relations”, as will be described below. The CTR method asks of the performers to inhibit their desire to act spontaneously and, instead, to exercise mindful decision-making as they take turns in improvising, using their bodies and props, to create artistic compositions. Performers are not to operate in an “instantaneous” or objective time, but a personal and relative “Real Time”, which is what we see as a Bergsonian time of the here-and-now (Bergson and Andison 2010). There is no mechanistic causality between past and present actions performed—actions just “are” for the sake of being. From a semiotic perspective (Peirce 1955 [1902]), the material generated in the improvisation is meant to be based on iconic and indexical relations, where symbols, narration, and narratives are excluded not only linguistically, but also creatively, in the materials used during the performance. A by-product of Fiadeiro’s method is learning to “understand and rethink decision, representation, and cooperation, both in art and in life” (Fiadeiro n.d.).

Because of the ethical implications of the method, the CTR has become of interest to people outside of the performing arts, from the fields of anthropology, economics, and complex systems (Fernandes and Jürgens 2016; Jürgens et al. 2019; Ribeiro and Evola 2017; Ribeiro et al. 2016). The CTR Game, initially intended for performers using their bodies on a stage floor, was adapted to a scaled-down version using props on a table during his 2011–2014 collaboration with anthropologist Fernanda Eugénio (previously called “AND_Game”; see Eugénio and Fiadeiro 2015). This gamification made the CTR more accessible to non-performers, unaccustomed to using their bodies in a performative environment, yet interested in practicing Fiadeiro’s methods.

The CTR improvisation exercise, a performance in its own right, may seem foreign to spectators unaccustomed to postmodern and contemporary dance^2^: Practitioners sit around the Game Table and silently, through means of self-selection, perform a single action at a time on the Game Table with props taken from the Objects Table to develop compositions. The movements and actions of each performer with their props builds on previous performers’ actions, with the table serving almost as a stage. There are only three rules: (1) only one performative act at a time per practitioner, (2) the immediately preceding act cannot be “corrected”, and (3) no act can be explicitly explained or justified. Throughout the improvisation, in fact, performers do not solicit or exchange any type of verbal information unless they are using their speech as creative material for the composition. More specifically:

Players use various objects to sequentially perform single actions, or “*positions*”, [so as to] create connections between positions. These connections are called “*relations*”. A player needs to perform a position which can have a relation (obvious to the other players) with the previous relation (connecting the previous two positions), and not with the previous position alone. Relations are built when a position has some sort of connection with how the Game has been played up to that point (in terms of size, color, placement, etc.). […] By being aware of what the environment offers and how potential personal contributions generate tendencies in relations already offered by that environment, participants are trained to coordinate their actions for a more consequent, consistent, and responsible overall performance, rather than impulsively performing an action. They are asked to always “take a step back” before taking a decision, and not to project their visions of how the Game should continue, but simply to comply with the previous actions. (Evola and Fiadeiro 2015)

Although a silent performance, there is social communication: turns are coordinated by information which is “given” (e.g., starting to move towards a prop) and information which is “given off” (e.g., via gaze and other potentially interpretable body movements) (Goffman 1963). The improvisation performance, much like language, is a symbolic system, where the purpose of each bodily action is “understood to be understood” across performers. It is a socially organized and coordinated activity situated in a shared perceptual space, which can be manipulated (Clark 2005). It involves embodied minds in a process of world-making (Goodman 1978). However, unlike with language, the turns are exclusively self-selected, and there is no explicit verbal communication involved.

### Coordinated Collaboration and Nonverbal Social Interactions

The interest in studying regulative and cooperative processes governing natural and spontaneous human–human interactions have a longstanding tradition in many scientific disciplines (e.g., sociology, psychology, and linguistics). The collaborative nature of conversational partners has been primarily investigated in the turn-taking system, firstly described by Sacks et al. (1974), with research on various contributing paralinguistic factors (e.g., speech rate: Manson et al. 2013; Street 1984; speech intensity: Huber 2008; Natale 1975; pitch: Local and Kelly 1986; intonation: Ford and Thompson 1996; breathing: McFarland 2001; Rochet-Capellan and Fuchs 2014; Włodarczak and Heldner 2016). For more practical reasons, most of this research has been concentrated on dyadic interactions (e.g., Kendon 1967), and more recently in multiparty situations (Bohus and Horvitz 2011; Petukhova and Bunt 2009). Research on coordinated collaboration has been done on turns in contexts where speech is absent, primarily in Deaf communities, where body-related strategies cueing turn self-selection have been evidenced (e.g., raising a hand from a rest position, changing head position or body posture, indexing with the hands, producing subtle noises such as tapping the table, and other overtly attention-getting signals: *inter alia* Baker 1977; McCleary and Leite 2013; van Herreweghe 2002).

Body movements have been cited as contributing to the coordinated process of social collaboration in a variety of ways. Turn-taking research indicates that people deploy a broad scope of body movements to yield or take the floor, such as pointing gestures (Goodwin 2000; Mondada 2007; Sikveland and Ogden 2012), head movements (Cerrato and Skhiri 2003; Duncan 1972; Hadar et al. 1985; Malisz et al. 2016; Rahayudi et al. 2014), eye gaze (Bavelas et al. 2002; Bavelas 2005; Brône et al. 2013; Jokinen 2009; Peters et al. 2005), prevocal preparations like mouth openings (Streeck and Hartge 1992), and body posture (Holler and Kendrick 2015). Beyond turn-taking, evidence shows that body movements when coupled with speech (co-verbal gestures, such as iconic and other representational gestures; Mittelberg and Evola 2014) provide information not present at the speech level which is successfully decoded by the observer–listener (Kendon 2015; McNeill 1992), even during speech-gesture mismatches (McNeill et al. 1994).

Body movements with no semantic meaning have also been shown to convey inferential meaning. Head turns and gaze shifts have a social function of cueing attentional shifts (Posner 1980). Postural mirroring and mimicry of other body movements have been cited as indicators of social collaboration (Oertel et al. 2013) versus social competition (LaFrance 1985; Newtson 1994), and can be spontaneously coordinated (see Fowler et al. 2008, for a review on bodily sways and posture). Self-adaptors, those body movements such as scratching or fidgeting typically produced under more cognitively taxing conditions (*inter alia* Ekman and Friesen 1969), not only have self-focused functions (e.g., acting as an aid to attentional focusing by “shielding” from distracting stimuli: Freedman and Grand 1985; contributing to better performance in attention tasks, like the Stroop test: Barroso et al. 1980), but they also convey inferential information to observers, affecting the social interaction (Chawla and Krauss 1994; Freedman et al. 1978).

Eye movements are particularly of interest when investigating coordinated collaboration and social interactions in general, in that gaze has a dual function in social cognition: to glean external information and to convey internal information (Abele 1986; Argyle and Cook 1976; Gobel et al. 2015). As much as any other body movements, movements of and around the eyes can communicate to another person some information either intentionally (as with deictic gaze) or inferentially (e.g., how intent the person is attending to something). It is, in fact, well established that gaze direction, gaze contact, and mutual gaze are ways in which humans, as well as other animals, convey information which is socially significant, signaling hostility or threat, joint attention, and the importance of objects in the environment, and the ability to interpret and predict other’s actions (Argyle and Cook 1976; Baron-Cohen 1994, 1995). In a collaborative setting, it aids in sustaining shared attention and establishing common ground (Brône and Oben 2015; Oertel et al. 2012; Rossano et al. 2009 for a survey). Gaze behavior and blinking reveals to which extent partners are engaged in their interaction (Cummins 2012).

### Silent Collaborative Coordination and the Body in Performing Arts

Research on the role of the body in coordinated collaboration in the performing arts where speech is not involved has gained increasing interest among scholars using data from musicians and dancers (using conductors’ data: Braem and Bräm 2001; Kumar and Morrison 2016; Poggi 2002). Research of this type, however, has either focused on singular types of behavior (e.g., formally involving particular articulators, or functionally serving particular purposes), using data of already “rehearsed” body movements which are consequentially more monitored and conventionalized in their context, or has been concentrated on the aesthetic and communicative impressions the performers’ body behavior has on an audience (e.g., using data from conductors: Kumar and Morrison 2016; musical improvisations: Moran et al. 2015; dance: Camurri et al. 2004; Kim 2016; using neurocognitive data: *inter alia* Bläsing et al. 2012, 2018; Pollick et al. 2018; Sevdalis and Keller 2011). In unrehearsed settings, data of performers’ interactions in jazz improvisations have been used for coordinated collaboration analyses mainly driven by the performers’ verbal feedback and interviews of their behavior, referring to the musical structures produced, and not the immediate behavioral data (inter alia Mendonça and Wallace 2004). Alessandro Duranti, unlike many others, draws from extemporaneous nonverbal data, such as gaze and other body movements (e.g., deictic gestures, foot tapping), as indicators of collaborative coordination among jazz performers in his analysis of intentionality and intersubjectivity in jazz improvisations (Duranti 2012, 2015, 2016; Duranti and Burrell 2004; see also Duranti and McCoy in press). With regard exclusively to dance improvisation, the body has mainly been treated on a macro-level (e.g., Torrents et al., 2016). Its involvement in collaborative coordination has been analyzed in terms of force dynamics and intersubjectivity, where the body participates in communicating intentions to the dance partner (Kimmel 2009), and in terms of the semiotic and phenomenological aspects of the bodies (Brandt 2015).

To our knowledge, this current study is the first to offer a systematic, fine-grained analysis of an entire set of performers’ gaze and other body movements in the collaborative coordination setting of the performing arts, specifically dance improvisation. The behavioral data collected and described is of a creative collaboration setting where speech is prohibited as a communicative tool, focusing on differences in body behavior between expert performers and non-performers during a contemporary dance improvisation exercise. A microanalysis of participants’ body movements will indicate what (types of) information is “given” or “given off” by the individuals. What is construed as meaningful by group members is based on their behavioral responses when coordinating less-typical social interactions in a creative collaboration.

## Hypotheses

As was described in the two preceding sections, previous research recognizes that natural and spontaneous social interactions rely on various nonverbal cues to coordinate collaboration, both with and without the co-presence of speech. More or less monitored bodily movements, ranging from gaze shifts to manual gestures to body posture, potentially communicate to others intentional and/or inferential meaning, such as turn-taking, attention-getting, and goal-oriented actions. Besides social functions, some body movements may also have an epistemic function for the producer, such as those movements to make an object more visibly accessible or to gain more information about a new environment.

In the case of the silent improvisation exercise, the main question of our inquiry is how expert performers communicate and coordinate collaboratively in dance improvisations without the use of language (*pace* intuition). This specific improvisational method (the CTR: see above) is regularly practiced by both performers and non-performers, often with equally creative outcomes; hence, there is an epistemological validity and legitimacy in comparing and contrasting the behavior of experts and non-performers. Still, the quality of the performance of professionals “feels” qualitatively different than a non-expert’s or non-performer’s, because of differences in how they move, which is quantifiable via formal features of the individuals and behavioral aspects of the social interaction.

This study will test a number of graduating hypotheses, the first testing a common belief which has not been investigated, namely that performers are more controlled in how they move. One way this can be expressed is:

1. H1: Performers produce fewer body movements overall compared to non-performers.

With no language involvement, the locus of all information production during an improvisation performance is the performer’s body, with its movements and its interaction with the environment. In any social interaction, the very presence of bodies in a shared space communicates something, and potentially available communicative information is in the bodies, whether they are moving or not (Bavelas 1990; Burgoon and Hoobler 2002; Goffman 1959). Performers and non-performers alike share the set of everyday strategies to communicate nonverbally, although performers also have gained the skill and awareness of not “stealing the stage”, so it follows that:

(2) H2: In terms of quantity, performers generate less intentionally communicative information than non-performers.
(3) H3: In terms of quality, performers’ communication-focused body movements will be more discrete in size and duration (using smaller articulators like eyebrows, short head movements, etc.).

Possibly the most inconspicuous body movements are the ones produced during gaze shifts, which are fundamental during silent interactions and have the social function of conveying inferential information (Argyle and Cook 1976; Baron-Cohen 1994, 1995; Brône and Oben 2015; Cummins 2012; Oertel et al. 2012; Rossano et al. 2009). Non-performers are expected to produce more gaze shifts to glean more contextual information (from the environment and from other co-participants’ actions) to make sense of the unconventional collaborative coordination setting, to which Expert Performers are already accustomed:

(4) H4: Performers’ gaze behavior production is more conservative than non-performers’.

Gaze also has the social function of conveying intentional information to an observer (Argyle and Cook 1976; Bavelas 2005; Rogers 2013; Shepherd 2010). We expect that Expert Performers, much like the Non-Performers, will rely on common gaze strategies during the improvisation as a discreet way of communicating and coordinating the collaboration:

(5) H5: Performers rely on gaze contact and mutual gaze as a channel to communicate and coordinate their actions during the collaborative flow (managing turns).

In contrast, it follows that Non-Performers produce more communication-focused movements as well as other more self-focused ones. They will exhibit a greater production of head turns and gaze shifts to gain more ambient and social information and to communicate their intentions as well as to seek approval, exercising more gaze contact and mutual gaze, typical of more common and familiar social settings. We speculate that Expert Performers and Non-Performers share the same set of human communicative nonverbal strategies which they use in everyday social interactions (see literature review above), but Performers have gained other cognitive and embodied dispositions which they employ when they are performing. If so, these differences will be manifested in their behavior.

Although instinctively the data should confirm these hypotheses, some interesting discrepancies appear, as will be described in the results and the discussion sections below.

## Method

### Participants

The BlackBox Real Time Composition (CTR) Floor Study investigates nonverbal coordinated collaboration across three different populations: professional performers who are also expert CTR practitioners (“Experts”); professional dancers naive to CTR (“Dancers”); and a third group of participants with no background in performing arts (“Non-Performers”). CTR Experts and Dancers had at least 8 years of professional dance/performance formal training and experience. CTR Experts additionally had on average 3 years of formal training and experience in Fiadeiro’s method and were selected from a pool of his professional collaborators. Non-Performers had no prior on-stage dance or performance experience of any kind. Each group was comprised of five participants plus the choreographer, resulting in a total of sixteen participants. The current paper will address only data from the Experts and Non-Performers groups, leaving the comparison with the Dancers data for a later analysis.

Taking socio-cultural factors into account, participants were screened and chosen so that each group was culturally mixed (Portuguese and non-Portuguese) and that each participant knew at least one other, but not all participants in the group. Furthermore, only participants proficient in English were selected to accommodate the common working language, despite the Game performances being silent.

The three groups were made up of five participants (3 females in the Expert and Dancer groups, 2 females in the Non-Performer group) plus the choreographer, and participants were between the ages of 26–41. Handedness was measured using the Edinburgh Handedness Inventory (Oldfield 1971); all participants were right-hand dominant with the exception of one Expert (3rd left decile).

### Setup and Equipment

**Fig. 1:**
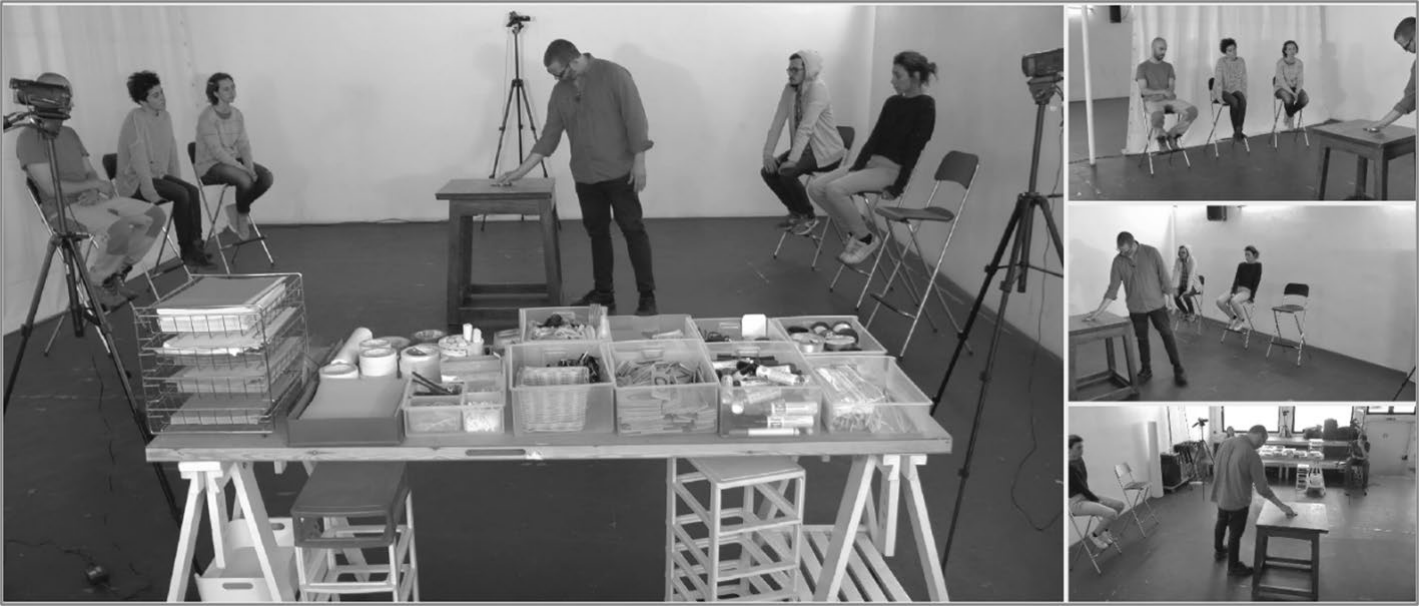
Camera perspectives highlighting setup and position of participants around the Game Table and the Objects Table (choreographer Fiadeiro at the Game Table)

In order to maintain as natural a setting as possible, the Floor Study was conducted at the RE.AL Atelier, Fiadeiro’s dance studio in Lisbon. The six participants per group were seated approximately 1.5 m away from and around the Game Table, which was the focal point of the Game performance (see Fig. 1). Props to be used during the improvisation exercise were provided by the choreographer (stationary material and other household items) and were readily available on the Objects Table. The scenario was captured from four different camera angles at 50 fps with full HD using progressive scanning: two video cameras (Panasonic HC-X920) were focusing on three participants on each side at a full-shot angle (head to toe), and the remaining two cameras were placed to frontally record the actions either at the Object or the Game Table at a wide angle. Two Filmgear Fresnel lighting units (1000 W) were used for better image quality. The choreographer was equipped with a lavalier microphone (Olympus LS-20M) to capture any spoken comments.

### Study Procedure

Before conducting the BlackBox Floor Study proper, two separate pilot sessions were conducted to test study conditions and validate the study procedure, including the annotation scheme to be used (data and material published in Evola et al. 2019). Study conditions were maintained across groups. Each group session lasted 4 h: briefing (30 min), introduction and Q&A by Fiadeiro (30 min), Game performance (2.5 h), and debriefing (30 min). A brief manual, prepared by the first author under the supervision of the choreographer (Evola and Fiadeiro, 2015), was sent to each participant prior to the study to guarantee a common basic knowledge of the CTR terminology and procedure across the groups.

After having been informed and having given consent, all participants were asked to review the Game guidelines and had a 30-min introduction from the choreographer. During this time participants were encouraged to ask for clarifications and to discuss the method and exercise via examples, followed by a practice trial (the Expert group did not use this total allotted time). Each group then had 2.5 h to perform several Games, with breaks managed by the choreographer, who provided feedback regarding the various actions in relation to the CTR. The final 30 min of each session were dedicated to further informing participants of the study, as well as to gathering their impressions about the Game and the CTR method.

### Data

The video data collected in the BlackBox Floor Study for all three groups totaled 10 h 14 s (with briefing, debriefing and breaks between Games). Here we only report on the Experts and Non-Performers groups’ sessions, and more specifically their performances of the Game, lasting 1 h 49 min 45 s. Given the lack of resources to annotate this amount of multiparty data, it was decided a priori to conduct the microanalysis on a minimum of the first 10% of this Game performance data, yielding the first 6 min of Game performance in each group. The frame-by-frame video annotation (Kita et al. 1998; Seyfeddinipur 2006) of all participants’ body movements in both Experts and Non-Performers groups equals 12 min, which should not seem insignificant considering the quantity of annotations the data produced (*n* = 3397).

It should be noted that there was a quantitative difference between the two groups in the time spent performing their first Game (Experts 21 min 23 s; Non-Performers 4 min 55 s). This meant that while the first 10% of Game performances (6 min) was taken from the beginning of the first Game of the Experts, in the case of the Non-Performers it was obtained by adding their entire first Game (4 min 55 s) together with the first 1 min 05 s of their second Game performance. The results section will contain analyses of the entire data subset (6 min), with two analyses focused on the first Game (4 min 55 s).

All audio and video data was pretreated to guarantee proper synchronization of recordings coming from the various devices using PluralEyes 3.5 and PremierPro CC 2015, and coded in ELAN 4.8.1 (Lausberg and Sloetjes 2009) using the annotation scheme described below.

### Annotation Scheme

Preexisting multimodal data annotation schemes were taken into consideration but were deemed unsuitable for this study (*inter alia* Baesler and Burgoon 1987; Bressem et al. 2013; Kipp et al. 2007; Kita et al. 1998; McNeill 1992). The majority of available coding schemes focus on co-speech gestures, or “utterance dedicated visible bodily actions” (Kendon 2015), where the objects of study are those body movements (especially manual ones) that humans more or less spontaneously produce accompanied by speech. This definition of “gesture” excludes other self-focused body movements produced without an intended socio-symbolic function, such as fidgets and other self-adaptors, which nonetheless may convey accidental information to who sees them. Moreover, available annotation schemes do not incorporate other body movements, such as gaze and directedness behavior (such as interaction with objects or movement in space), which often are not systematically included in the coding process and only glossed when highly relevant. Furthermore, these schemes have been mainly devised intending to look at data of humans interacting in conversations where the subjects remain seated in close proximity. In contrast, the nature of the data collected from dance improvisation and other performing arts contexts is chiefly nonverbal (e.g., Camurri et al. 2004; Duranti 2016; Kimmel 2009), spontaneous speech is often suppressed for performance reasons, and the bodies are more dynamic in space.

Nonetheless, some features of these annotation systems have been adopted in the present scheme (such as preparatory movements and indices). The NEUROGES annotation system (Lausberg 2013; Lausberg and Sloetjes 2009), in our view, does stand apart from others for its inclusion of the kinetic coding of articulator movements (principally hands) and its successive coding of these formal movements in functional terms. This idea of initially coding all movements (versus non-movements) in all body articulators was inspired in part by this system and gave rise to the construct of “movement unit” (see below: Annotation cluster 2, Formal description of movement units). It should be noted that, while this term was used earlier by Kita et al. (1998) to describe body movements spontaneously produced in the context of language, our definition applies solely to physiological movement.

The coding system used in this study was created, tested, and validated in-house (see validation details below). The annotation manual included detailed instructions, such as video rate viewing for each tier, definitions, and other specifics for the frame-by-frame segmentation and labeling. The data from each participant was annotated individually in separate files on twelve tiers in the ELAN video annotation software, using templates and controlled vocabulary to avoid gross errors. A “game_action” tier temporally segmented the participant’s self-selected turn to perform an improvised act in the Game (coded from the onset of preparatory movement until the onset of a default pose at their home base). A speech tier was included for the eventuality of any speech or vocables (see Results and discussion: Speech and vocables), as was an operational “notes” tier for any rater observations. The remaining nine tiers were divided into three annotation clusters: The first two clusters have a more objective nature than the third. This last cluster of annotations is more prone to a social interpretation, that is, to how other observers might meaningfully interpret a given body movement in that particular context.

#### Annotation Cluster 1: Directedness Behavior

The first order of coding was to annotate all participants’ directedness behavior. This means coding to which co-participants, objects, or actions the participants were oriented, in particular their intentional, or purposeful actions. Three tiers coded for:

- spatial location/orientation and body posture (sit, stand, at_table, home, goTo_prep; goTo_X, settling, etc.);
- gaze end-points (gaze_to_P2, gaze_down, gaze_to_table, etc.);
- and object interaction (get, hold, use, release, etc.).

This cluster of annotations is objective in nature, aiming to computationally track where the bodies are at all times and to what people are attending. It should be noted that the object interaction tier has been largely excluded from the present analysis and reserved for future research.

#### Annotation Cluster 2: Formal Description of Movement Units (MUs)

The second cluster of our annotation scheme targets solely at the kinematic movements of body articulators. Annotations were segmented for each articulators’ movement unit. Regardless of any communicative meaning in these movements, the scheme refers to which articulators are activated (i.e., moving vs not-moving) in three distinct regions:

- head/face (head, eyes, head + eyes, mouth, head + mouth, etc.);
- upper body (hand_left, arm_right, shoulders_both, up_limb_left, torso_back, etc.);
- and lower body (foot_right, legs_both, lb_limb_right, hips, etc.).

We define a *movement unit (MU)* as a gestural complex marked by the distinct change of an articulator’s configuration or position in space either from the default configuration/position or with respect to a previous MU. An MU can be monophasic (e.g., a hand shift) or multiphasic (e.g., head nods, shakes). The onset of the MU was marked from the first clear frame where an articulator configuration was detected as changing either from the default position (as described in each section of the coding manual) or with respect to the previous MU. The offset of the MU was marked at the last frame of the movement and/or the final hold of that movement before either going back to a default position or changing to a different configuration, indicating the beginning of a new MU. Furthermore, only those body movements produced by muscular tension in that articulator were annotated (following Özyürek 2000; Lausberg 2013).

#### Annotation Cluster 3: Functional-Semiotic Taxonomy of MUs

Each segment of the MUs’ formal description had assigned to it one of three interpretational functions: self-, context-, and communication-focused.

1. *Self*-*focused movements* are physical movements that are meant for the self, having no relation or representation for the outside world. They are essentially biological self-adaptors. Whether these gestures are more or less monitored [like scratching an itch or “gesturing-for-thinking” (Kita 2000) while manipulating an imaginary object] or not (like fidgeting), they are solely focused on and destined for who is performing them. Personal objects (e.g., glasses, jewelry) are considered as an “extended-self” and interaction with these also were coded as self-focused movements. Other examples include body shifts, irregular blinking, yawning, and some head tosses.
2. *Context*-*focused movements* are relational movements, in that they establish a physical or cognitive relation (orientation, attention, volition, etc.). These include glances and gazes, orientational head-turns (for directed attention), and action-oriented movements (moving towards something, picking something up). These movements have an epistemic function of knowing what, where, how things are, or what, where, how one is with respect to other people or things. Other examples include: leg tension in preparation to get up, scanning and tracking with the head, and deliberate attention-gaining actions.
3. *Communication*-*focused movements* are representational movements, which have a symbolic and social nature. These are destined to be interpreted by another person. Often characterized by gaze co-presence, these include, for example, intentional yawning to communicate boredom, smiling to communicate agreement, and raised eyebrows to express surprise.

This functional taxonomy is a semiotic hierarchy, based on Peircean relations of firstness, secondness, and thirdness (Peirce 1955 [1902]), with gradually increasing complexity, where the higher order builds on (and includes) the lower one(s). By virtue of this hierarchy, if a body movement could be coded as having more than one function, in that the coder had a strong indecision between one or another category, the higher was always selected (e.g., a person leaning forward in their chair could be coded as self-focused, but ultimately if the person was getting up from their chair to do something, this would be coded as a context-focused movement). Semiotically speaking, any body movement could be inferred and construed as having a communicative function, even if the person making that movement does not have the intention of communicating that. For example, we may infer that a person fidgeting is nervous, but that would not necessarily make that action a communication-focused one (unless it was staged deliberately to convey that sense). Nonetheless, our analysis is obviously not from the production perspective, as we do not have immediate access to what the participants were thinking while they were performing these body movements. An interpretation perspective allows us to focus on how these movements were socially construed by others, and from this interpretative and hermeneutic perspective, whether or not a certain movement’s primary focus was intended to be communicative (in a strict sense), notwithstanding what information can be inferred. The high inter-rater agreement allows us to deduce that the observing study participants would statistically also agree with how these movements have been interpreted.

### Inter-rater Agreement and Data Reliability

Two main steps were taken to ensure that the annotation scheme was reliable and to validate the processed data. First, two raters (the authors) independently tested a beta version of the annotation scheme against a 3-min sample of pilot study data, which led to critical discussions and revisions regarding how to better define the segmentation and labeling of the annotations (e.g., excluding or merging redundant labels, validating categorical labels by removing any “undetermined” or other catch all labels).

Second, this revised version of the coding scheme was used with a 3-min sample of video data from a second pilot study, again independently by the two coders. Inter-rater agreement from this annotated data was calculated using the measurement of the modified Cohen’s kappa (Holle and Rein 2013) built into ELAN. The values obtained for each tier separately ranged between *κ* = 0.6471 and *κ* = 1, consistent with “substantial” to “perfect agreement” (Landis and Koch 1977; reports of the individual kappa values can be provided upon request). Thus, these results indicated that the developed annotation scheme was a sufficient and valid measurement instrument to use on the actual Floor Study data.

This final version of the annotation scheme was then used by the two independent raters to initially code 50% of the Expert data subset (i.e., three of the six participants), with the inter-rater reliability calculated resulting chiefly in “almost perfect agreement” (Table 1). These high values of agreement ensured that one coder (first author) could proceed confidently with the annotation of the remaining 50% of the Expert group data, and that annotating the Non-Performers’ data could be divided among the two (with second author annotating the tiers for gaze and head/face MU and function).

**Table 1:** Inter-rater agreement values obtained in the CTR Game study data from two raters coding data from three participants (P), or 50% of the Expert Performers’ dataset

	Global results of inter-rater agreement		
	Kappa	Kappa max	Raw agreement
P4	0.6352	0.7590	0.6500
P5	0.9041	0.9281	0.9111
P6	0.9516	0.9516	0.9541

As a final word addressing potential biases of the authors of the study executing the data coding, it is important to highlight that the coding was performed on a frame-by-frame basis (50 fps) and that the categories (save “functions”) involve highly nondiscriminatory categories. To take for example the author-raters’ potential bias of seeing one group producing more movement units than another (see H1). At the very basis of the coding scheme is distinguishing movement versus non-movement of participant’s various body articulators, focusing first on those in the head/face region, then in the upper- and lower-body regions separately, comparing one frame with the next at each stage. Detecting a frame-by-frame image difference of this kind is highly objective and does not involve extra-perceptual evaluation. Inter-rater disagreements are more likely to occur with regards to which exact frame a movement begins or ends, but less likely as to whether there was movement or not (see Holle and Rein 2013). As a frame of reference: if a rater was biased that one group should produce less movements than the other, while coding a one-second movement, that rater needed to be stubbornly biased 50 consecutive times (for each second), as 50 different frames must be evaluated to detect presence or absence of movement. The bias of that rater would translate into disagreement with a second rater, yielding a low kappa coefficient value. Finally, having the main authors of any study participate in the coding of the data allows them to have a more intimate understanding of the data rather than having a collection of decontextualized results. This is an ulterior advantage, when the proper methodological care has been taken and when the research allows it, granted annotation between raters is independent and blind.

## Results and Discussion

### Speech and Vocables

The CTR improvisation exercise is a silent exercise, in that practitioners are instructed not to talk during the improvisation performance. In the data from the Expert Performers group, there were 0 s of speech or vocables, in contrast to the Non-Performers data, which includes 10 s 13 ms of dialogue between participants.

The fact that Non-Performers did use speech is not interpreted as a bug in the data (i.e., invalid data caused from a misunderstanding of the task), but as evidence of a natural tendency of non-performers to elicit and exchange information using whatever resources they have available.

### Body Movement Units: Formal Annotations

The data subsets from the Expert Performers (EP) and Non-Performers (NP) groups resulted in a total of 3397 annotations from the game action tier and the tiers of the three annotation clusters (described in “Annotation scheme” above; Table 2). The data from the Expert Performers’ group yielded 1196 annotations, almost half as many than the Non-Performers’ (*n* = 2201), confirming (H1). Experts performed marginally fewer game actions (EP: *n* = 10 vs. NP: *n* = 12), changing their posture and location in space 38% less often than Non-Performers (EP: *n* = 96 vs. NP: *n* = 156). Gaze shifts occurred some 35% less frequently in the Experts group (EP: *n* = 448 vs. NP: *n* = 691). The data from both groups confirms that most body movements are produced in the head/face region and using hands as is described by other research (see Harrigan et al. 2005). However, movements produced by facial articulators and the head were about half as many in the Experts as in the Non-Performers data (EP: *n* = 141 vs. NP: *n* = 330), with a similar trend for those generated in the upper (EP: *n* = 122 vs. NP: *n* = 220) and lower body (EP: *n* = 58 vs. NP: *n* = 121).

**Table 2:** Comparison of the distribution of annotation counts across the nine tiers between the Expert Performers (EP) and Non-Performers (NP) groups

Tier	Annotations (*n*) per group	
	EP	NP
Game action	10	12
Location/posture	96	156
Gaze	448	691
Head/face		
Movement unit	141	330
Function	141	330
Upper body		
Movement unit	122	220
Function	122	220
Lower body		
Movement unit	58	121
Function	58	121
∑	1196	2201

*EP* Expert Performers, *NP* Non-Performers

### Body Movement Units: Functional Annotations

One of three functions was assigned to each formally annotated MU in both groups (EP: *n* = 321 vs. NP: *n* = 671) in order to test (H2-H3). Movement units having a communication-focused function were more infrequent than those with context- and self-focused functions both in and between groups (Table 3).

**Table 3:** Frequency of movement functions between the two groups, distributed per body region where the MU was produced

Body region	MU function					
	Self-focused (*n* = 670)		Context-focused (*n* = 302)		Communication-focused (*n* = 20)	
	EP	NP	EP	NP	EP	NP
Head/face	76	105	65	206	0	19
Upper body	109	209	13	10	0	1
Lower body	57	114	1	7	0	0
∑	242	428	79	223	0	20

#### Communication-Focused MUs

Communication-focused MUs were absent in the Expert group data (*n* = 0), as opposed to the Non-Performers (*n* = 20), chiefly produced in the head/face region (*n* = 19) and once involving the entire upper body (*n* = 1). The most frequent MUs in the head/face area were produced with head (*n* = 8) and mouth (*n* = 6), and other subtypes had fewer instances: head and face (*n* = 1), head and only mouth (*n* = 3), and eyebrows (*n* = 1).

Communication-focused MUs are defined as representational movements, having a symbolic and social nature destined to be interpreted by another person. The hypothesis that there be fewer communication-focused movements in the Expert group than the Non-Performers group not only is confirmed in our data, but the data goes as far as to indicate zero instances of nonverbal (or verbal) intentional information being conveyed, which supports (H2) and renders (H3) moot. As for the Non-Performers, their communicative intentions were literally written on their faces: articulators in the head/face region were overwhelmingly used to convey intentionally communicative content. One explanation for this is that the Non-Performers fell back on those nonverbal social interaction strategies commonly used in conversation, whereas the Expert Performers have embodied other strategies that the Non-Performers do not have available. This suggests that any collaborative coordination between the Expert participants occurred on a different level than via speech or overtly communication-focused movements in the head/face, upper or lower body. In other words, the Expert Performers avoided using intentional communication, relying on information to be inferentially communicated in order to coordinate collaboratively.

#### Context-Focused MUs

Context-focused MUs were the second most frequent functional category, with the Expert data having about a third of the occurrences of the Non-Performers’ (EP: *n* = 79 vs. NP: *n* = 223), mainly in the head/face region (EP: *n* = 65 vs. NP: *n* = 206) because of orientational head-turns. In both groups, only a small number of movement units in the upper body was labelled with the context-focused function (EP: *n* = 13 vs. NP: *n* = 10), involving either the entire upper body (i.e., both limbs plus torso; EP: *n* = 5 vs. NP: *n* = 6) or just the torso (EP: *n* = 3 vs. NP: *n* = 3). The data shows only one instance of context-focused MU in the lower body produced by an Expert participant (*n* = 1), when the participant uncrossed her legs to initiate a game action (contra NP: *n* = 7).

Besides communicative-focused movements (absent in the Expert data), context-focused movements are those most relevant and useful in the social interaction of coordinated collaboration. Context-focused movements have the function of establishing a physical or cognitive relation between the subject and her environment; any communication of information is accidental to the degree that it is perceivable by others. In terms of information processing, context-focused movements in social interactions have a dual function: epistemic for the producer, and hermeneutic for the observer. In terms of theory of mind, they serve the observer as inferential cues of goal-directedness of an intentional agent (see Carruthers and Smith 1996), eliciting the former to behave accordingly (like, among cooperative performers, not to steal the floor). In the context of this study, context-focused movements produced in the head/face region could only inferentially mean the decision-making process was still being elaborated (for example, via head turns); those produced elsewhere (especially hands, limbs) could be interpreted by the observers as part of a decision-*taking* process, or in other words, as a behavioral expression of engaging with the environment and within the social and collaborative context.

The need to frame the context and to gain more information from their surroundings, including their co-participants, may have prompted more head turns among Non-Performers. Expert Performers, on the other hand, exerted more control over not making superfluous movements which could be inferred as being socially relevant, like attentional shifts. The inferential information relevant to the collaborative coordination among Expert Performers varies, from preparing to take a turn (placing the hands on the seat with a stiff upper body in preparation to stand up) to not taking a turn (sitting on one’s hands, or sitting back against the chair). The data indicates that the majority of these movements were produced using the torso and or all articulators of the upper body, meaning that these movements are marked and highly visible to observers. Context-focused movements in the upper body are the only ones that Experts produced proportionally more than Non-Performers—marginally more in terms of counts, but meaningful when comparing to the fact that they consistently otherwise produced less movements. This supports the claim that Expert Performers have acquired a greater awareness of the inferential information conveyed by (their own and others’) body movements, that they minimize context-focused movements, and that their stillness is the intentional information they aim to communicate.

#### Self-Focused MUs

Self-focused MUs were produced chiefly in the upper body region (EP: *n* = 109 vs. NP: *n* = 209) in and between groups, and most frequently using the hands (EP: *n* = 58 vs. NP: *n* = 156). Instances of bimanual self-focused movements among the Expert Performers were 91% fewer (*n* = 7) compared to Non-Performers (*n* = 78). Whereas hand dominance for self-focused MUs was almost perfectly balanced in the Experts data (*n*_LH_ = 25; *n*_RH_ = 26), the Non-Performers data yielded twice as many right-handed than left-handed self-focused movements (*n*_LH_ = 26; *n*_RH_ = 52). Self-focused MUs in the head/face region occurred 28% less often in the Experts’ data (EP: *n* = 76 vs. NP: *n* = 105), and in both groups mainly involving the head, either alone (EP: *n* = 41 vs. NP: *n* = 46) or together with some facial articulator (EP: *n* = 9 vs. NP: *n *= 11). The second most prominent articulator in this region is the mouth, alone (EP: *n* = 16 vs. NP: *n* = 34) or together with the head (EP: *n* = 7 vs. NP: *n* = 8). Self-focused MUs in the lower body region follow the trend of the Expert group data of having fewer occurrences than the Non-Performers (EP: *n* = 57 vs. NP: *n* = 114). When a bilateral articular was involved in the upper- and lower-body regions, the data indicated a slight preference of left- over right-sided articulators in both groups (EP: *n*_*left*_ = 43, *n*_*right*_ = 41 vs. NP: *n*_*left*_ = 74, *n*_*right*_ = 71).

The idea that variations in emotional states, like being cognitively taxed or relaxed, cause variations in muscular tension dates back to the mid-Twentieth century and has been studied since then (*inter alia* “autistic movements”: Krout 1935; Sainsbury 1955; “self-adaptors”: Ekman and Friesen 1969). Self-focused MUs were the most frequent category in both groups, making up 75% of all Expert MUs and 64% of all Non-Performer MUs. One explanation for the lower proportion among Non-Performers is that they were engaging in relatively more communication-focused and social behavior. An alternative and perhaps complementary interpretation is that although Expert Performers were able to control their body movements in performance mode, these more spontaneous movements are controllable only to a certain degree.

### Gaze

Eye-gaze data are reported in terms of counts (number of gaze shifts) and duration (time between gaze shifts). Table 4 compares these results of both groups shifting their gaze to any co-participant, to the Game Table (focal point of the exercise), to the Objects Table (with available props), or elsewhere (looking down, up, free, etc.). A total of 1139 gaze annotations was identified in the entire data set, with the Experts shifting their gaze 35% less frequently than Non-Performers (EP: *n* = 448 vs. NP: *n* = 691). Less frequent gaze shifts is a strategy commonly used to plan future actions during high cognitive load visuomotor tasks as opposed to storing the perceptual information in working memory (Ballard et al. 1995; Perdreau and Cavanagh 2015).

**Table 4:** Description of eye-gaze data produced during each group’s dance improvisation exercise. “Other” incorporates annotation categories like gaze up, down, free, etc

Group	Gaze target	*n*	Total duration (s)	Mean (s)	SD (s)
Expert Performers	Game Table	148	1262.122	8.53	13.0
	Object Table	40	139.466	3.49	2.72
	Co-participant	166	387.228	2.33	7.58
	Other	94	371.191	3.95	8.78
Non-Performers	Game Table	215	1093.639	5.08	6.19
	Object Table	101	330.466	3.27	4.84
	Co-participant	297	551.350	1.86	2.39
	Other	78	76.523	0.98	0.70

In both groups, more gaze shifts were directed towards co-participants than any other target category (EP: *n* = 166 vs. NP: *n* = 297), with the second most frequently looked at target being the Game Table (EP: *n* = 148 vs. NP: *n* = 215). In contrast to their Non-Performer counterparts, the Experts tended to shift their gaze least towards the table with props (EP: *n* = 40 vs. NP: *n* = 101), looking more towards neutral spaces (up, down, free, etc.; EP: *n* = 94 vs. NP: *n* = 78).

In terms of gaze duration, both groups spent the majority of their time looking towards the Game Table, with the Experts looking slightly more (EP: 1262.122 s vs. NP: 1093.639 s) despite comparatively fewer gaze shifts. In both groups, the second-most time spent gazing was towards a co-participant, with the Experts’ total duration being 30% less (EP: 387.228 s vs. NP: 551.350 s). Despite a relatively balanced number of gaze shifts towards the “other” category, the Experts group spent almost five times more time gazing towards these more neutral spaces than Non-Performers (EP: 371.191 s vs. NP: 76.523 s); whereas the latter spent about that same amount of time directing their gaze to the table with the props (EP: 139.466 s vs. NP: 330.466 s).

Results from the gaze data confirm (H4) and reinforce (H1). In general, the data indicates that the Expert Performers were in more control of all their body movements, including eye movements, shifting their gaze less frequently than Non-Performers and mainly focusing on the Game Table, where the performance was happening. They avoided looking at the table with the props (Objects Table), which could act as a cue to their co-performers of anticipating a turn-taking, and preferring to gaze into neutral spaces around them. Bearing in mind the sociocognitive function of gaze among humans, the results of gazing to co-participants is particularly relevant. Experts spent 30% less time looking at co-participants than the Non-Performers who were monitoring more what their co-participants were doing.

#### Mutual Gaze

Mutual gaze, where any two participants look at each other reciprocally, amounts to 1119 ms (Min = 159 ms, Max = 516 ms, Mean = 373 ms, Median = 444 ms, SD = 189 ms) in the Experts’ data, with three distinct occurrences, two of which take place within the first 30 s of the Game. These can be more properly described as glance exchanges, in that they lasted on average 373 ms. These data are compared to the Non-Performers’ group, which had 10 instances of mutual gaze totaling 6516 ms, each lasting on average 652 ms (Min = 41 ms, Max = 1780 ms, Mean = 652 ms, Median = 364 ms, SD= 646 ms).

The most surprising finding in the Expert Performers’ data is the near lack of mutual gaze, fundamental for joint-attention (Rogers 2013), which was expected in a silent, collaborative coordination context where turns need to be managed, hence refuting (H5) but reinforcing (H1) and (H2). The data, however, have more indications of mutual gaze activity among Non-Performers, with longer duration times. All but two instances of Non-Performers’ mutual gazes were between a participant and the choreographer. One explanation for this is that Non-Performers were seeking approval or confirmation from the authority figure by means of gaze contact. An alternative, complementary interpretation is that the choreographer has adapted himself to the context, behaving less like a performer. In fact, the choreographer is not involved in any mutual gaze with the Experts.

Mutual gaze was most present at the beginning of the coordinated collaboration. All instances of mutual gaze among Non-Performers occurred within the first 60 s, as well as two of the three instances of mutual gaze between four Expert Performers happening within the first 30 s of the improvisation exercise. Although the participants had already spent considerable amount of time together, these instances of mutual gaze at the beginning of the improvisation exercise serve almost as a “visual handshake”, a sign of recognition and establishing the shared perceptual field of interaction (Duranti 1997) of the performance.

### Temporal Relations

#### Temporal Distribution of Gaze Activity

**Fig. 2:**
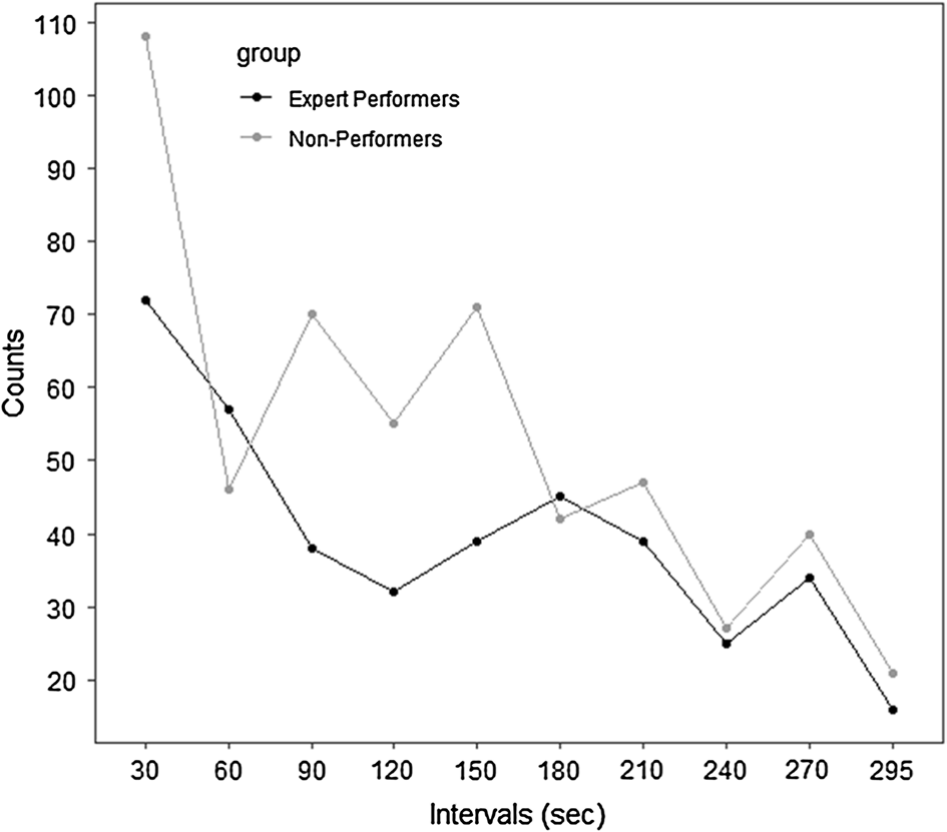
The distribution of eye-gaze activity in 30-s intervals between Expert Performers and Non-Performers groups

When considering the distribution of gaze shift frequencies in 30-s intervals (see Fig. 2), there is a trend of decreased gaze-shift activity after the first 30-s in both groups, and within the first 2 min the number continues to considerably decrease (EP: − 56%: vs NP: − 49%). Moreover, the number of gaze shifts are never as high as within the first 30-s in both groups. The Experts begin with more controlled gaze behavior than the Non-Performers, and there appears to be a trend of Non-Performers’ gaze behavior with time being more similar to the Expert Performers’ from 180 s into the exercise. The slope-intercept form was calculated to establish the change in the number of occurrences between second 30 (t_1_) and 180 (t_2_) in each data subset:

$$ y = mx + b,\quad {\text{where}}\quad m = \frac{{n_{1} - n_{2} }}{{t_{1} - t_{2} }} $$

A lower degree of slope variability was observed in the Expert Performers’ than in the Non-Performers’ data. Whereas the slope of the Expert group decreased only 37.5% (*n*_1_ = 45, *n*_2_ = 72), the slope of the Non-Performers’ group declined 61.1% (*n*_1_ = 42, *n*_2_ = 108). In other words, much as is anecdotally described in meditation practiced by experts and non-experts, the Non-Performers do reach a behavior similar to that of the Experts, but requiring more time. The Expert group was quicker in reaching a more consistent gaze behavior; however, like the Non-Performers, there was a disproportionally high gaze activity within the first minute of the task. Studies using gaze data, especially in more social contexts, could take into consideration that the first 1 or 2 minutes of gaze activity may skew results of the data.

#### Temporal Relationship Between Gaze Shifts and Movement Units

**Fig. 3:**
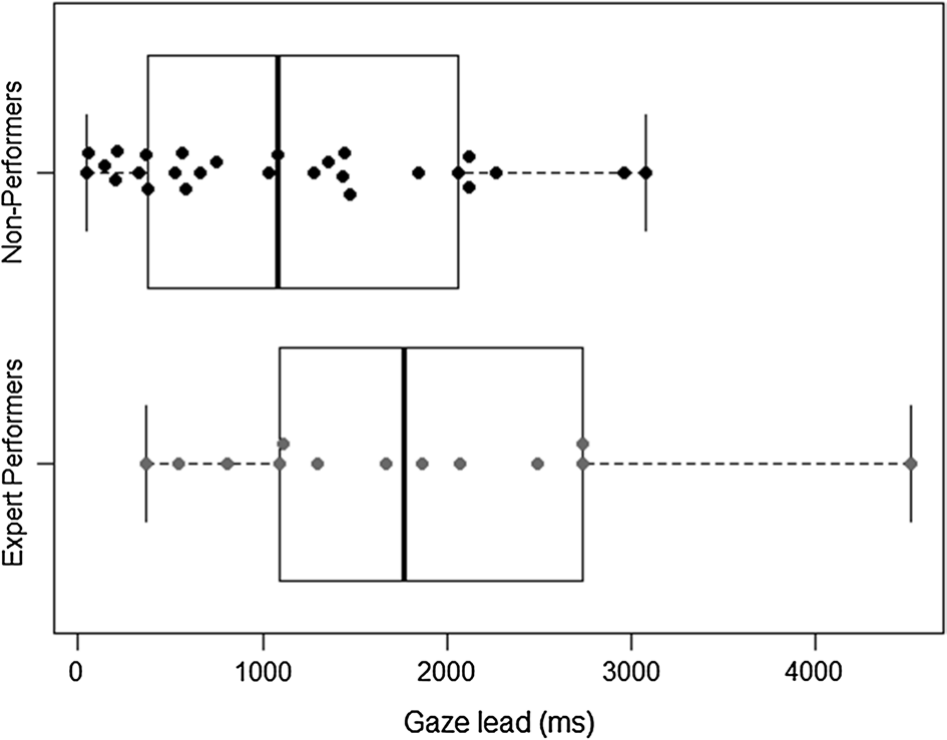
Temporal relationship between gaze and movement units (MU) annotations. The lead defines the distance between (a) the MU onset of any co-participant and (b) the successive onset of participants’ gaze shifts towards that moving co-participant. Three outliers are not represented (EP: 18 s; NP: 5.6 s and 6.7 s)

Because gaze shifts are indicative of attentional shifts, which can be triggered by movement in the field of vision, we investigated the temporal relationship between (a) participants’ gaze shifts towards a moving co-participant with (b) the overlapping movement units of the latter. Instances of temporal distance between the MU onset of any co-participant and the successive onset of participants’ gaze shifts towards that moving co-participant were plotted (Fig. 3). A total number of 43 time-aligned gaze-to-MU segments was identified during the first Game in both groups (EP: *n* = 14 vs. NP: *n* = 29). The average lead duration varies in the subsets. Whereas the Non-Performers shifted their gaze to moving co-participants one second after they started moving (Mean = 1472 ms, Median = 1083 ms, SD = 1558 ms), the Expert Performers waited twice as long before looking towards the moving co-participant (Mean = 2947 ms, Median = 1083 ms, SD = 4455 ms).

One interpretation is that the Experts have an understanding that not all movements in their field of vision have inferential or intentional meaning, and using endogenous control (Posner 1980), they wait longer before shifting their gaze to a co-participant making a movement of any type. On the contrary, the Non-Experts are seeking for any available information about the coordinated collaboration, thus shifting their attention more readily.

## Final Remarks and Conclusions

Choreographer João Fiadeiro is fond of saying that everyone is a dancer whether they know it or not, a sentiment shared by many today in the field of dance (Stinson 1999). When asked after the improvisation exercises how he would rate the performances between expert and non-performers, he replied that they were equally as interesting: both performance exercises were successful, and in fact participants in both groups were very creative with their compositions according to Fiadeiro. Nonetheless, as outside observers, there is a feeling of “performance” when watching experts that is not there when watching non-performers practicing the same improvisation exercise. In a follow-up evaluation study, 31 participants were asked to view and assess the collective behavior of the blinded groups (e.g., hesitant, decisive, graceful, awkward). Interestingly enough, when participants were asked to rate each group’s collaborative behavior independently on a Likert-type scale, there was no significant difference; however, in self-reported comments which compared the two groups side-by-side, the Non-Performers were described as more communicative (talking and exchanging gazes), but also as “disconnected”, “indecisive, guessing” and “hesitant”. On the other hand, the Expert Performers were described as “individualistic”, “pensive” and “thought-through”, “seemed to know better what kind of behaviour was ‘expected’”, and even “choreographed”.

Although everyone is a dancer, not everyone has a dancer’s “intuition”. This intuition might be better described as “skilled decision-making”, stressing more the nature of it being something acquired and learned rather than something inside everyone. With relation specifically to improvisation performance and CTR practitioners, they are experts in controlling even minor movements with the knowledge that these might affect how the performance develops. Skilled decision-making and performance collapse in “Real Time” for the performer, “in a single flow of awareness of how their presence and their actions fit together with the presence and actions of the others in a cohesive developing pattern of relations” (Leland McCleary: personal communication).

The results of this study indicate that expert performers have acquired specialized skills in using and exhibiting their body during the coordinated collaboration of an improvised performance. The expression of these skills, first and foremost control over socially meaningful body movements, is evidenced by the comparison of behavioral data with non-performers sufficiently trained to perform the CTR method exercise. The tools that experts have during a collaborated coordination are not different from those of non-performers, but it is the way they use them which differs. As experts in the use of the body, performers have become more aware of the tools they have available, together with their affordances and limitations, and how to use them appropriately for the specific purpose of the social interaction which is performance.

With the understanding that a body in space, be it still or in motion, communicates information, expert performers generate less intentionally communicative body movements (including with their eyes) so as not to produce distracting signals during a performance. Goffman (1959) noted that, through “impression management”, people are in fact able to control what information they “give off” (to a certain extent, as seen above) in a variety of social interactions. In very broad terms, performers enter a “performance mode”, avoiding unnecessary movements which might distract co-performers and audience so as not to “steal the stage”, a strategy not acquired by non-performers, who interact with a “noisy body”. Compared to the Non-Performers group, which served as a control, the Experts produced about half as many movements of any type, small or large, and with any articulator. In fact, the number of movement units they produced with individual or a complex of articulators were between 47 and 59% less depending on the body region.

Performers’ use of silence and stillness is a form of intentional communication and is socially meaningful for collaborative coordination. The most surprising finding in our data was that the Expert Performers were coordinating their collaboration successfully, despite not using speech, producing zero instances of communication-focused movements, avoiding gaze contact, including a near absence of mutual gaze, normally considered fundamental for joint-attention. We believe that Expert Performers exploit their peripheral vision to recruit contextual and social information, which is supported by the data of the temporal relationship between participants’ gaze shifts and co-participants’ movement units as well as our observations of blinking patterns of the video data (not reported here). To detect turn-taking, parafoveal and peripheral vision is sufficient to detect movements, such as someone getting out of her chair to perform. Covert attention (Carrasco et al. 2000; Hunt and Kingstone 2003) is presumably activated by the Expert Performers as a strategy while they are fixating on the Game Table without ostensibly and unnecessarily moving their heads. In other words, the Expert Performers intentionally allocate their attention following the goal or task they have at hand. This endogenous orienting (Posner 1980) requires broadening the scope of perceptual attention, which in turn, may affect creativity by generating more original and extra-categorical uses for the Game objects in this improvisation (Friedman et al. 2003).

Gaze is often perceived as an autonomous behavior with the eyes moving to whatever attracts them. As our data indicate, gaze, in fact, is often controlled and monitored by the person according to the context. In the case of performing arts, gaze becomes an important part of the social interaction context, and much like metaphors and co-verbal gestures, it is a “structuring structure” (Bourdieu 1977) which is embodied as part of the embedded social agent’s conceptual system (Evola 2010a, b, 2012). Our data indicate that performers use gaze as a specific embodied practice, in the more sociological term, besides its more common socio-cognitive use of gleaning external information and conveying internal information. Characteristic of this secondary habitus in our data is gaze avoidance, which is not only motivated by the desire to be focused on the creative production during the improvisation. Refraining from recognizing co-performers during the improvisation (via gaze contact) serves a social function of displaying that they are participating in that very practice of performance, and that they are adhering to what is expected of an expert performer in that context. Paradoxically, by avoiding social recognition during the performance, they are being connected with their dance partners, who expect that type of behavior, hence forming a coordinated collaborative and communicative behavior.

## Footnotes

1 Video data and other supplementary material accompanying this article can be found at http://blackbox.fcsh.unl.pt/JONB.html (see also Evola et al. 2019).

2 More information about the choreographer João Fiadeiro as well as video examples may be accessed from: http://blackbox.fcsh.unl.pt/joao-fiadeiro.html.
